# Fishbone as a Foreign Body in the Pharynx - CT Density for Prediction of Fishbone and the Type of Fish on the Western Coast of Karnataka

**DOI:** 10.1007/s12070-025-05614-1

**Published:** 2025-06-18

**Authors:** Santosh Rai PV, Rajesh Nayak, Akshada Atchut Kauthankar, Deval Rishi Pandit, Pareekshith Raghupal, Nishith Shetty, Arathy Mary John, Vijendra Shenoy

**Affiliations:** 1 Department of Radiodiagnosis and Imaging, Kasturba Medical College Mangalore, Manipal Academy of Higher Education, Manipal, India; 2 Department of Otorhinolaryngology and Head Neck Surgery, Kasturba Medical College Mangalore, Manipal Academy of Higher Education, Manipal, India

**Keywords:** Computed Tomography, Fish Bone, Foreign Body, HU Unit

## Abstract

**Background:**

Fishbone is a widespread foreign body accidentally swallowed, accounting for about 84% of ingested foreign bodies. Most of the patients do not present with any symptoms. The common sites where foreign bodies get embedded in the upper gastrointestinal tract involve the base of the tongue, the palatine tonsils, the pyriform sinus, and the vallecula.

**Methods:**

The Department of Radiodiagnosis and Imaging, Kasturba Medical College Hospital, Mangalore, conducted a cross-sectional study involving CT examination of seven types of commonly consumed fish in the coastal Karnataka region, before and after cooking. After the scan, the ROI was drawn on the skull, hypural, spine, rib, upper jaw, and lower jaw to measure the HU unit for all seven fish.

**Result:**

The study reported that the average HU unit of the skull, hypural, spine, rib, upper jaw, and lower jaw of fish before and after boiling. The mean value of skull bone showed 195.4 ± 141.7 and184.9 ± 123.2. The mean values of 284.1 ± 191.8 and 279.7 ± 105.7 for the spine. Similarly, for the hypural 242.1 ± 117.4 and 170.7 ± 100.8. For the ribs, the mean values were shown as 114.1 ± 59.8 and 99.4 ± 49.4. The average value of the upper jaw was 251 ± 198.9 and 116.6 ± 110.7.

**Conclusion:**

The study provides an idea of the different ranges of HU value in the fish commonly consumed in the coastal Karnataka region. This information can aid in detecting the fish bone foreign bodies in patients with a suspected history of fishbone impaction.

## Introduction

Ingestion of foreign bodies is a common indication for hospital visits. Most of these ingested bodies transit the gastrointestinal tract without any difficulties [1]. Of all the foreign bodies, accidental ingestion of fish bone (FB) is by far the most common and accounts for 84% of accidentally ingested items [2]. The common sites where foreign bodies get embedded in the upper gastrointestinal tract involve the base of the tongue, the palatine tonsils, the pyriform sinus, and the vallecula [3]. Another common site is the cervical esophagus [4]. 

Most ingested fish bones exit naturally without any issues, while about 10–20% of cases may not pass. The linear, fine, and sharp structure may cause the bone to adhere and infiltrate the mucosa, which can result in severe complications [5]. Complications from the same have been noted in less than 1% of the cases which can include inflammatory changes, formation of abscess, perforation of the mucosa, bowel obstruction, and hemorrhage [3, 6]. 

Diagnosing the presence of fish bone on plain X-ray films has a low sensitivity of 25–39% but a high specificity of 91% [2]. Computed Tomography (CT) on the other hand offers 100% sensitivity and 93% specificity [7]. Factors such as the size of the bone, the place of bone impaction as well as the radiodensity which changes with different fish species affect the visibility of the bone [8]. Contrast-enhanced CT scans can cause impede the identification of fish bones [2]. 

The purpose of this study was to investigate the Hounsfield Units (HU) of the fishbone in diverse types of fishes found commonly along the western coast of Karnataka to aid in early detection of fishbone foreign bodies in the upper Gastro-intestinal tract and suggest a diagnostic protocol.

## Methodology

**Fig. 1:**
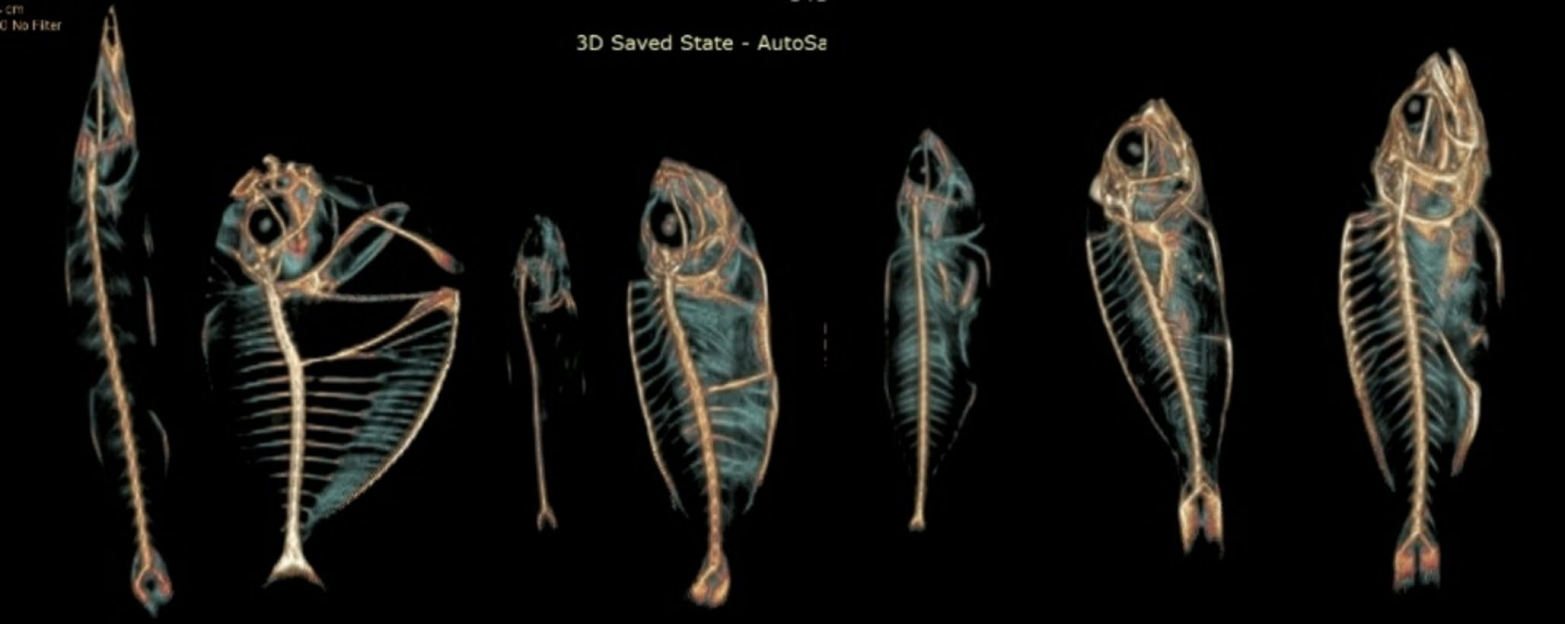
3D images of the before-boiling fish

**Fig. 2:**
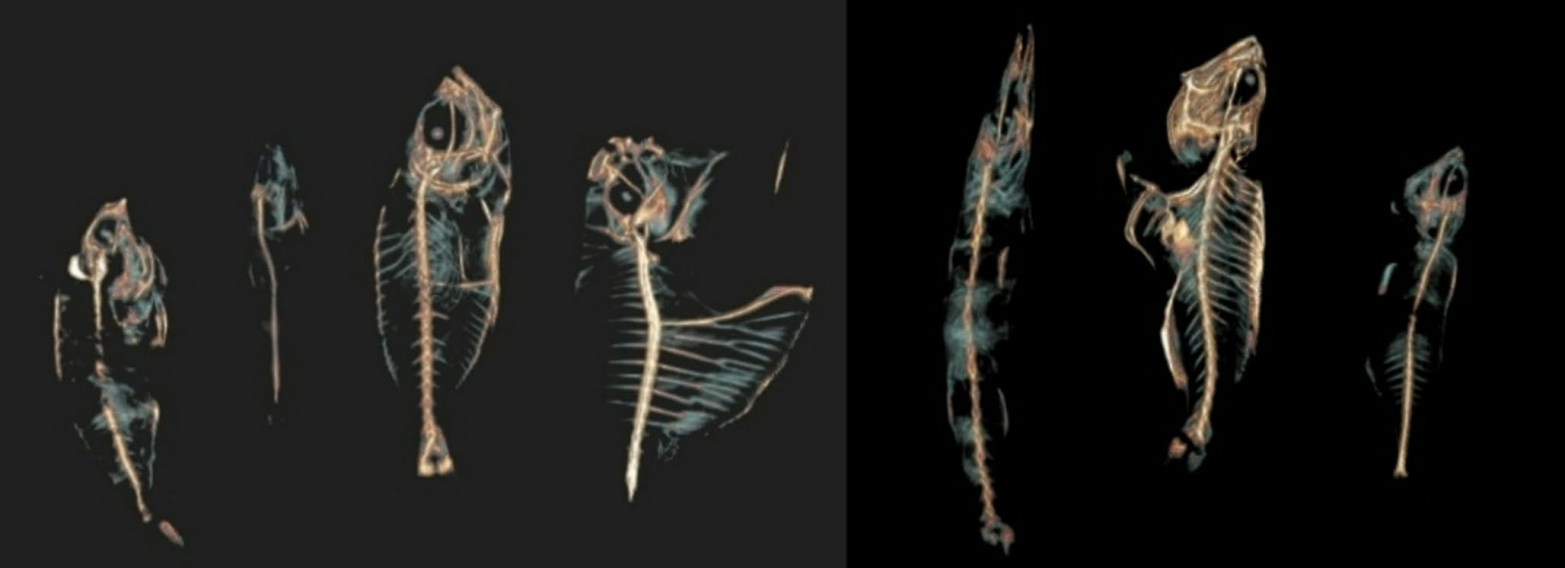
3D images of the after-boiling fish

The Department of Radiodiagnosis and Imaging, Kasturba Medical College Hospital, Attavar, Mangalore, conducted a cross-sectional study involving seven types of commonly consumed fish in the coastal Karnataka region. These included ladyfish, ponyfish, pink perch, finned bullseye, sardine, mackerel, and reef cod. Since the study involved the commonly consumed fish, no ethical clearance was obtained. The standard Indian and the local names in Karnataka are listed in Table 1. The fish was placed in the radiolucent tray, and a CT scan was performed before and after cooking the fish using a 16-slice Brivo General Electric Computed Tomography scanner with a 1 mm slice thickness. These images were converted into three-dimensional (3D) images, as shown in Figs. 1 and 2. The ROI was drawn on the skull, hypural, spine, rib, upper jaw, and lower jaw to measure the HU value for all seven fish in the axial slices.

### Statistical Analysis

The statistical analysis was performed using the Jamovi 2.3.28. Descriptive statistics were used to determine the fishbone’s mean and standard deviation before and after cooking.

## Result

Seven different types of commonly consumed fish in the coastal Karnataka region were included in the study. These included Ladyfish, ponyfish, pink perch, finned bullseye, sardine, mackerel, and reef cod.

**Table 1:** Fish names

Scientific name	Common name	Kannada name/ Local name
Nemipterus japonicus	Pink perch	Madmal, Rani
Rastrelliger kanagurta	Indian Mackeral	Bangude, Bangada
Sardinella longiceps	Oil Sardine	Boothai, Mathai
Leiognathus equula	Pony fish	Kurichi, Kaaral
Epinephelus diacanthus	Reef cod	Muru, Panna, Kalava
Elops saurus	Lady fish	Kaane, kalan
Priacanthus hamrur	Finned bulleye	Disco fish

**Fig. 3:**
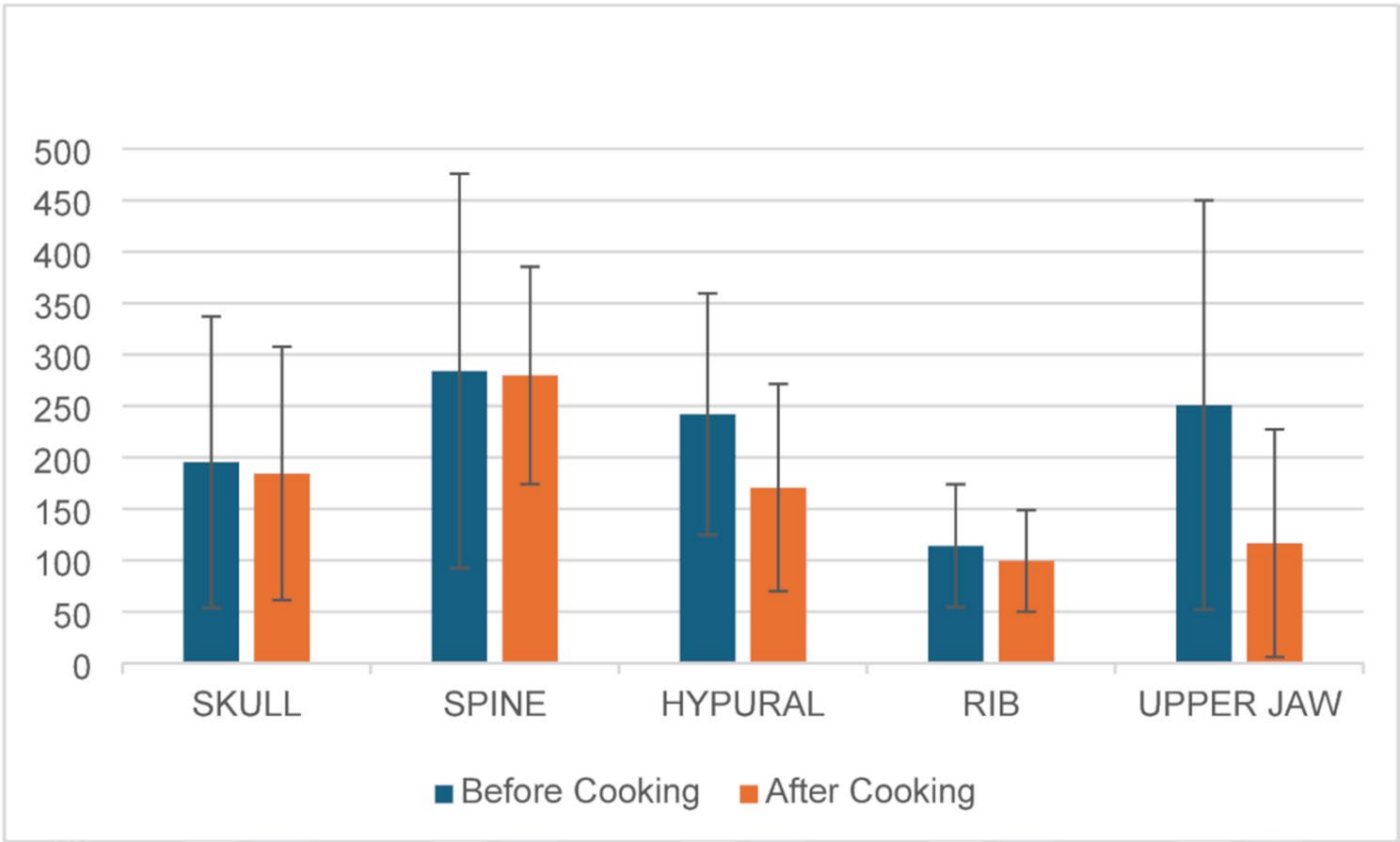
Mean of HU unit. The standard deviation is represented in error bars

The study analyzed the HU unit of the skull, hypural, spine, rib, upper jaw, and lower jaw of fish. Descriptive statistics are shown in Table 2; Fig. 3. The study reported that the skull bone of the fish showed a mean value of 195.4 ± 141.7 before boiling and HU of 184.9 ± 123.2 after boiling. Before cooking the fish, the pink perch and mackerel showed a maximum and minimum HU units of 458 & 23 respectively. Similarly, after boiling, the sardine and pink perch showed minimum and maximum HU units (18 & 347). The pink perch showed more HU units before and after cooking.

The mean values of 284.1 ± 191.8 and 279.7 ± 105.7 for the spine were observed before and after boiling. Similarly, the mean values of hypural were shown as 242.1 ± 117.4 and 170.7 ± 100.8 before and after cooking. The maximum HU units of 649 and 436 were observed for the spine and hypural of pony fish. The mackerel fish and sardine fish showed a minimum HU unit (25 & 77) before cooking. After cooking the fish, the maximum HU units of 412 and 270 were shown for spine and hypural in the pony fish and mackerel fish, respectively. Sardine fish showed a minimum HU unit for the spine and hypural.

For the ribs, before cooking and after cooking, the mean values were shown as 114.1 ± 59.8 and 99.4 ± 49.4. Before and after cooking, maximum HU units (149 & 179) were detected on pink perch and reef cod fish. Similarly, the minimum HU unit was observed in sardine fish before and after cooking.

For the upper jaw of the fish, the maximum HU units of 581 were shown on pink perch before cooking, and after cooking, the maximum HU unit of 329 was shown on reef cod fish. The minimum HU units (24 & 17) were observed in the sardine fish before cooking and after cooking. The average value of HU units before and after cooking for the upper jaw was 251 ± 198.9 and 116.6 ± 110.7.

**Table 2:** Descriptive statistics for the before and after cooking the fish

Variables		n	Mean	SD	95% CI	
					Upper	Lower
Skull	Before cooking	7	195.4	141.7	326	64.4
	After cooking	7	184.4	123.2	299	70.9
Spine	Before cooking	7	284.1	191.8	462	107
	After cooking	7	279.7	105.7	377	182
Hypural	Before cooking	7	242.1	117.4	351	134
	After cooking	7	170.7	100.8	264	77.5
Rib	Before cooking	7	114.1	59.8	169	58.8
	After cooking	7	99.4	49.4	145	53.7
Upper Jaw	Before cooking	7	251.0	198.9	435	67.1
	After cooking	7	116.6	110.7	219	14.2

*CI confidence interval, SD standard deviation, n Number

## Discussion

Fishbone is an extremely common foreign body that is accidentally swallowed, accounting for about 84% of accidentally ingested foreign bodies [9]. The majority of the patients do not present with any symptoms. However, a few may have sensations in the upper oesophagus. A patient with a history of eating a fish, complaining of constant darting pain in the esophagus is an indication of the possibility of a lodged fish bone. The FBs usually exit the gastrointestinal tract, and there is spontaneous resolution of symptoms. Common sites of fish bone impingement include the pyriform sinus, lingual and palatine tonsils, and the vallecula. Occasionally, the patient may present late after ingestion with serious problems including GI perforation, infection or abscess in the deep neck or mediastinum, or aorta-esophageal fistula. Perforation, obstruction, and abscess formation of the gastrointestinal tract may occasionally lead to death. [10–11, 12–6]

The current study was planned to find out whether non-invasive procedures such as CT morphology and CT density can predict fish bone density and the type of fish ingested when a fishbone as a foreign body is suspected in the pharynx and/or upper GIT. This will help in identifying the species and help in planning complete surgical procedures which may be endoscopic or open methods. Seven different types of commonly consumed fish in the coastal Karnataka region included in the study were Ladyfish, ponyfish, pink perch, disco fish, sardine, mackerel, and reef cod. CT scan was performed before and after cooking the fish with a 1 mm slice thickness. After the scan, the ROI was drawn on the skull, hypural, spine, rib, upper jaw, and lower jaw to measure the HU unit for all seven fish. CT scan was chosen as the modality of choice as it has been reported to have 100% sensitivity and is, therefore, the gold standard for diagnosing suspected upper GI foreign bodies that are not visible on plain radiographs.

Daniel Kim et al. [13] also recommended computed tomography to confirm the diagnosis of an impacted fish bone. Carr et al. studied frequently consumed fish in the UK which included skate, salmon trout, mackerel, salmon, and herring. The study found that several fish bones had low visibility on X-rays. The absolute investigation for identifying throat foreign bodies was through rigid endoscopy which is invasive and often associated with inherent risks and complications. In the preoperative diagnosis, clinical signs and routine radiography are not reliable in diagnosing fish bone impaction in the throat. This drawback was solved with the applications of CT, which has been reported to be more sensitive (~ 95%) than plain films in the detection of ingested fish bones although can be mistaken for normal structures or obscured by oral contrast [14]. 

In a prospective study by Woo SH et al. [15], a total of 286 patients with fishbone impaction were examined to determine the most effective imaging modality for identifying the fishbone. It was noted that routine radiography identified 35.9% of simple bones, while CT identified 54.5% of the gill bones. The majority of the fish bones were identified in the cervical esophagus (65 cases, 98.5%), with only a few found in the lower esophagus (1 case, 1.5%). The study concluded that CT is a valuable diagnostic tool and the primary method of choice for detecting foreign bodies in the esophagus. A retrospective study conducted by Kumar S et al. [16] demonstrated that X-ray versus CT had a sensitivity of 42.9% and 87.5% and a specificity of 73.3% and 71.4% respectively to identify upper aerodigestive tract fish bone impaction. The study inferred plain radiography may be insufficient to diagnose fishbone impaction and low-dose CT may be used for screening. Kirkam & English et al. [17] reported two fatalities caused by fish bones, lodging in the pyriform fossa and hypopharynx. Lateral neck X-rays were used to rule out fishbone presence in both cases. The sensitivity of lateral neck radiographs for directly visualizing fish bones is low, approximately 35% (ranging from 25 to 39%). Fishbones are frequently identified in the oropharynx in patients less than 40 years of age while in patients greater than 40 years, fishbones were identified in the esophagus. Plain CT is recommended in patients > 40 years old irrespective of the symptoms [11]. 

A study by Coulier et al. [18] highlighted the importance of slice thickness in CT to detect the foreign body. In the current study CT scan was performed using a 16-slice scanner before and after cooking the fish with a 1 mm slice thickness. In our study few of the studied fish bones as sardine before and after cooking were found to have the lowest HU density, so thin slices of CT may be useful for accurate diagnosis of those fishes with lower HU.

Goh B et al. [2] utilized CT for the preoperative diagnosis in patients with GI perforation following fish bone impaction. The authors observed that the scanning thickness of CT is a limiting factor. Thinner slices are useful in differentiating calcified foreign bodies from blood vessels. This approach also reduces the risk of missing a foreign body between slices, particularly when dealing with movement artifacts.

Liew CJY et al. [19] observed that on multidetector CT, fish bones typically appear either as linear, Y-shaped, or irregularly shaped radio-dense structures. These can measure between 1 and 3 cm in length and can be oriented in various directions. Imaging is crucial in cases of foreign body (FB) impaction, as it helps clinicians identify the exact location of the FB, which is essential for treatment planning. The shape and orientation of the FB are also significant, as vertically or linearly oriented FBs are generally easier to remove than horizontally impacted or irregularly shaped ones. Additionally, assessing the intraluminal component is important because clinicians usually attempt endoscopic removal of the bones.

Dilute oral contrast and CT can be helpful if the intraluminal component cannot be completely identified. Studying the density of the impacted fish bone using a CT scan can also be used to identify the species of fish ingested which can further help in planning treatment. In our study, the HU of fishes commonly consumed in Karnataka varied across the different parts of the skeletal structure. The highest HU was noted in the spine followed by the hypural, upper jaw, skull, and ribs before cooking. After cooking, the highest HU was noted in the spine followed by the skull, hypural, upper jaw, and rib. Overall amongst the different types of fish, the pink perch showed the highest HU while sardine showed the lowest HU, before and after cooking.

A retrospective observational study was conducted by Shishido T et al. [20] from October 2015 to May 2020 involving 368 patients to investigate the presence of fishbone in the upper aerodigestive tract. The most frequent fish that was observed was Eel followed by mackerel, salmon, horse mackerel, and flounder. The fish bones of the flounder fish were frequently trapped in the hypopharynx and esophagus (9 out of 30 cases, 30%), making spontaneous dislocation unlikely and often requiring surgical or endoscopic removal (19 out of 29 cases, 65.5%). The study concluded that identifying the fish species can aid in the accurate diagnosis and effective treatment. In a retrospective review by Feinmesser G. et al. [21] plain film neck radiography showed poor predictive capability for bone impaction (sensitivity 14.4%, specificity 89.8%, accuracy 63.2%), whereas CT scans exhibited significantly improved accuracy and sensitivity in detecting bone impactions (sensitivity 83.3%, specificity 94.1%, accuracy 92.5%). Specifically, neck X-rays did not detect 60 out of 61 oropharyngeal bone impactions.

By providing specific HU values for various fish types before and after cooking, this research enhances diagnostic accuracy, enabling clinicians and radiologists to better identify and differentiate fish bones from other organic materials, such as chicken bones, on CT scans. Understanding the precise HU ranges for different fish bones aids in detecting those that may be invisible during endoscopy or ambiguous on X-rays, while also offering insights into the type of fish ingested based on geographical region. Additionally, analyzing the density of the impacted foreign body, along with the type of bone and the level of cooking, can help predict the likelihood of injury to the aerodigestive tract and associated complications, such as perforation or infection, particularly in high-risk populations. This knowledge can also augment treatment decisions regarding whether to opt for endoscopic removal or conservative management, depending on the visibility of the bone in imaging studies. The limitation of the study is that it focuses on the commonly consumed fish in the coastal Karnataka region, whereas depending on geographic regions and cultures the tendencies in fish consumption and the methods of cooking may vary; therefore, the characteristics of fish-bone foreign bodies may also change. However, most of the fish species identified in this research, such as sardine, mackerel, and reef cod, are common globally. As a result, the findings of this research can be broadly tailored to diagnose fish-bone foreign bodies by calculating the density of fishbone on thin-slice CT. Use of dual-energy CT for color mapping based on fish remnant particles.

## Conclusion

Different varieties of fish have different densities in the various parts of their bony system. The current study provides an idea of these different ranges of Hounsfield units in the commonly consumed fish in the coastal Karnataka region. The highest HU was noted in the spine followed by the hypural, upper jaw, skull, and ribs before cooking. After cooking, the highest HU was noted in the spine followed by the skull., hypural, upper jaw, and ribs. Amongst the different types of fishes, the pink perch showed the highest HU while sardine showed the lowest HU, before and after cooking. This information can aid in the detection of the fish bone foreign bodies in patients with a suspected history of fishbone impaction.
